# Response to “Comment on ‘Independent associations of high‐density lipoprotein cholesterol and triglyceride levels with Alzheimer's disease and related dementias’ ”

**DOI:** 10.1002/alz.70234

**Published:** 2025-05-30

**Authors:** Erin L. Ferguson, Thomas J. Hoffmann, Akinyemi Oni‐Orisan, Neil Risch, Ronald M. Krauss, Catherine A. Schaefer, Maria Glymour

**Affiliations:** ^1^ Department of Epidemiology and Biostatistics University of California San Francisco California USA; ^2^ Institute for Human Genetics University of California San Francisco California USA; ^3^ Department of Clinical Pharmacy University of California San Francisco California USA; ^4^ Kaiser Permanente Division of Research Oakland California USA; ^5^ Department of Pediatrics and Medicine University of California San Francisco California USA; ^6^ Department of Epidemiology Boston University School of Public Health Boston Massachusetts USA

1

We thank Dr. Lin for their thoughtful comments on our manuscript, which evaluated independent associations of high‐density lipoprotein cholesterol (HDL‐C) and triglycerides with dementia risk.^1^ In addition to recognizing the clinical relevance of our work, they raised five points related to limitations and future directions. We address each point in turn.

First, as noted in our manuscript, we agree that this study is susceptible to unmeasured confounding, and this possibility must temper any causal inferences about the effect of cholesterol on dementia risk. While factors like frailty may confound these relationships, it is likely that these types of confounders would affect all types of cholesterol (HDL‐C, low density lipoprotein cholesterol [LDL‐C], and triglycerides) similarly. We therefore consider it notable that HDL‐C, LDL‐C, and triglycerides were estimated to have *different* associations with dementia risk, suggesting observed associations are unlikely to be entirely attributable to unmeasured confounders with similar effects across the lipid types.

Second, the present manuscript uses an average of all cholesterol measurements over a 2‐year period. We previously showed that using a single versus average measure of LDL‐C and HDL‐C did not meaningfully change estimates.^2^ We agree that our paper did not evaluate longer‐term changes in cholesterol. This is a different, important research question addressed in some other studies.^3^, ^4^

Third, statins are not likely to be a major mediator between HDL‐C or triglycerides and dementia risk. Statins largely affect LDL‐C, which we have shown is not associated with dementia risk.^2^ It is unlikely that patients would receive statins because of low HDL‐C or high triglycerides alone.

Fourth, while outside the scope of the present work, we agree there is potential for effect modification by cardiometabolic markers.

Fifth, we presented results for residualized lipid measures to better address internal validity. We also presented estimates for transformed (but not residualized) HDL‐C and triglycerides in Tables S3 and S7.

In conclusion, our paper found that low levels of HDL‐C and triglycerides in late‐life were each independently associated with dementia risk. Additionally, high levels of HDL‐C were not associated with dementia risk after adjusting for triglycerides. Clinical interventions targeting low HDL‐C and triglycerides could be important for dementia prevention if these relationships are causal.

## CONFLICT OF INTEREST STATEMENT

The authors declare no conflicts of interest. Author disclosures are available in supporting information.

## Supporting information



Supporting Information
